# Primary Calvarial Lymphoma: A Case Report

**DOI:** 10.7759/cureus.55210

**Published:** 2024-02-29

**Authors:** Mohammad G Abdoh, Balgees Ajlan, Abdulaziz A Basurrah, Sultan Al-Saiari, Syeddah S Mujtaba, Elham Rawah, Zaina Brinji, Abdulgadir Atteiah, Ahmed A Farag

**Affiliations:** 1 Neurosurgery Department, King Abdullah Medical City, Makkah, SAU; 2 Neurosurgery Department, Dalhousie University, Halifax, Nova Scotia, CAN; 3 Neurosurgery Department, King Abdullah Medical City, Mecca, SAU; 4 Pathology Department, King Abdullah Medical City, Makkah, SAU; 5 Radiology Department, King Abdullah Medical City, Makkah, SAU; 6 Neurosurgery Department, King Fahad Hospital, Jeddah, SAU

**Keywords:** b-cell, lymphoma, calvarial, skull lesion, biopsy

## Abstract

Calvarial lymphoma is radiologically similar in many respects to meningiomas, solid fibrous tumours, osteomyelitis, and metastatic carcinomas. Even though it is an extremely rare phenomenon, the initial suspicion and detection of calvarial lymphoma are paramount to establishing a correct diagnosis which helps to determine an appropriate management strategy.

We present an illustrative rare case of primary calvarial lymphoma along with a literature review focusing on the best management strategy for this rare entity.

A 45-year-old female presented to our center in March 2022. She had a history of forehead swelling, which was progressively increasing in size over time. The metastatic workup and bone marrow biopsy were negative. Initially, extensive surgery was planned to resect the lesion, but after a discussion with the multidisciplinary team, a biopsy of the lesion was taken, which revealed a large B-cell lymphoma.

It is prudent to consider calvarial lymphoma in the differential diagnosis of a progressively growing skull lesion, which may obviate the need for large resective surgery. A biopsy plus chemoradiation may be all that is required.

## Introduction

Invasion of bone by lymphoma is not in itself an uncommon phenomenon, but it is rare for lymphoma to arise primarily in bone [1]. Primary bone lymphoma represents less than 1% of all malignant lymphomas. A more typical location would be in the long bones of the lower extremities, spine, ribs, and pelvis [1]. Due to its rarity, it is not usually included in the radiological differential diagnosis of intracalvarial lesions. Major surgical procedures for calvarial lymphoma may not be the best modality. Although it is widely accepted that the use of adjuvant chemotherapy and radiation is necessary, the role of surgery, however, varies greatly depending on clinical settings [2].

Prior research has mainly put emphasis on the radiological and diagnostic features of calvarial lymphoma [3-12]. However, to our knowledge, very few studies have addressed management plans and outcomes. In this study, we add a new case of calvarial lymphoma to the literature and discuss further the role of surgery. We assume that when suspicion of calvarial lymphoma is high, a simple tissue diagnosis prior to chemoradiation is usually adequate for the majority of these patients without the undue risk of major surgery.

## Case presentation

**Figure 1:**
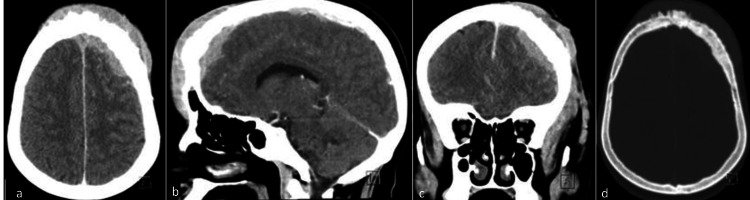
CT brain [a] axial, [b] saggittal, and [c] coronal images, and [d] an axial bone window showing a frontal, extra-axial, enhancing lesion, most of which is associated with expansion of the left frontal bone, periosteal reaction and extension to the overlying subcutaneous tissue.

**Figure 2:**
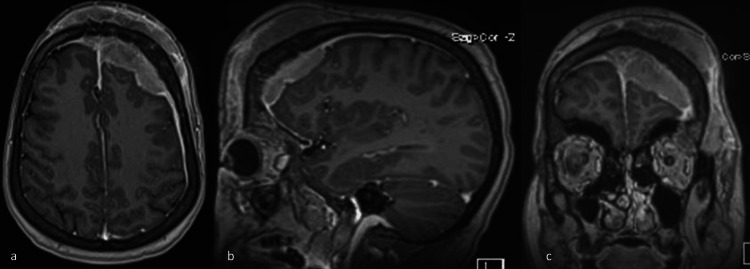
T1 contrasted MRI [a] axial, [b] saggittal, and [c] coronal images showing a frontal, extra-axial, enhancing lesion, extra-axial enhancing lesions, with extension to the overlying subcutaneous tissue. The lesion is occupying the superior sagittal sinus in keeping with invasion.

**Figure 3:**
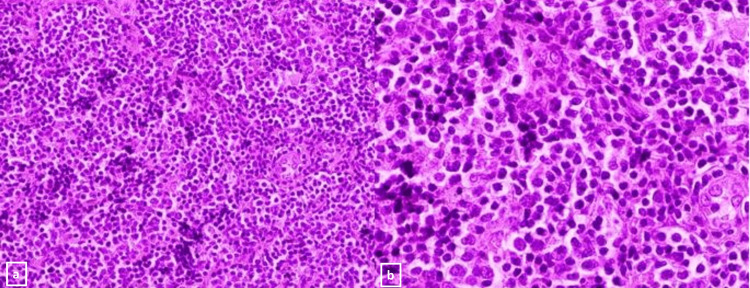
a, b - Hematoxylin and eosin stains at 20X and 40X respectively - Images show sheets of atypical/malignant lymphoid cells, large in size and having enlarged, round nuclei with vesicular and coarse chromatin pattern, conspicuous nucleoli and have moderate amount of pale cytoplasm.

**Figure 4:**
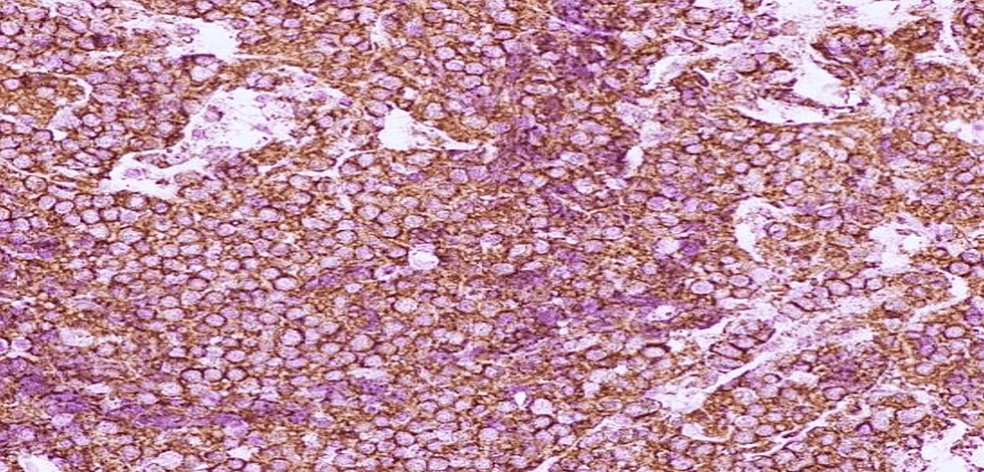
Lymphoma cells are found positive for CD-20 immunostain.

**Figure 5:**
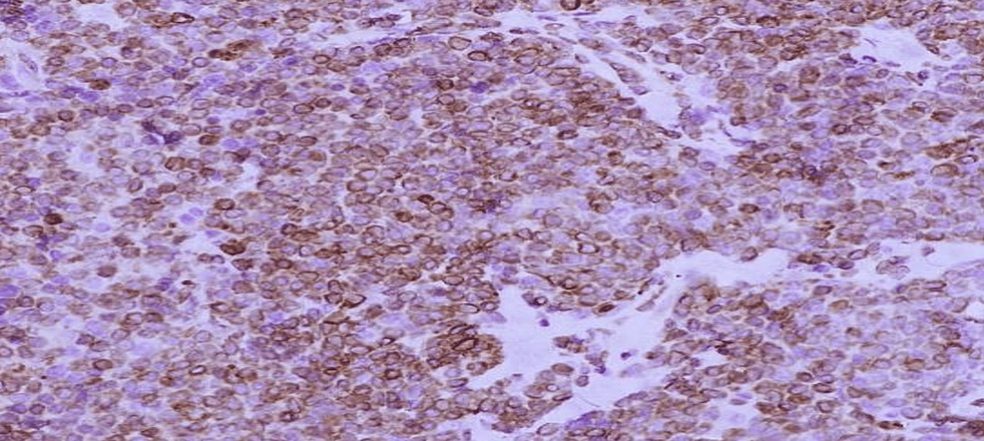
B-cell lymphoma 2 (Bcl-2) protein is positive in lymphoma cells.

**Figure 6:**
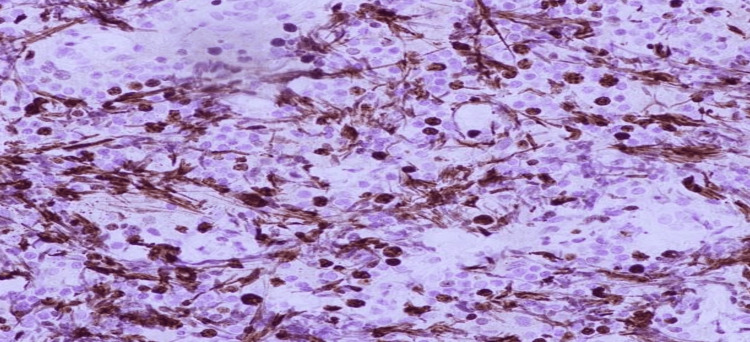
High Ki-67 proliferation index, approximately 50 to 60%.

A 45-year-old female was seen as a new referral to neurosurgery at our center in March 2022. She had a history of forehead swelling that had been progressively increasing in size over one year. She denied having headaches, seizures, memory issues, or weakness. She had occasional blurry vision in the left eye and was also complaining of left eye swelling and an inability to fully open the eye. She was diabetic and was on oral medication. She looked well but had swelling of the forehead and left side of the face, and proptosis of the eye. Radiological studies, including computed tomography (CT) and magnetic resonance imaging (MRI) (Figures 1, 2), and magnetic resonance venography (MRV) of the brain, revealed multiple midline extra-axial enhancing lesions associated with expansion of the left frontal bone and periosteal reaction with extension to the overlying subcutaneous tissue. The lesion was occupying the superior sagittal sinus with loss of normal flow, consistent with invasion. While these radiological findings suggested a malignant bone tumor, a sarcoma with a soft tissue component, or dural metastasis, an en plaque meningioma was a remote possibility. No abnormalities were found in the CT scans of the chest, abdomen, and pelvis, and the bone marrow biopsy was normal. Initially, our plan was to perform open surgery to remove the lesion. However, following a discussion in the MDT meeting, it was decided to begin with a biopsy of the lesion. A biopsy was taken from the soft tissue lesion of the frontal scalp with the patient under local anesthesia. The hematoxylin and eosin (H&E) stained slides of the biopsy showed the presence of a large lymphoma cell (Figure 3). Immunohistochemical stains, including CD-10, B-lymphoma protein 2, and Ki-67 proliferative index, were high (Figures 4-6), confirming the diagnosis of large B-cell lymphoma.

## Discussion

Calvarial lymphoma is an extremely rare phenomenon, and early suspicion and detection of it are crucial for establishing a correct diagnosis and determining appropriate management. Tissue diagnosis is needed to determine the proper management plan.

In this report, we have described the presentation, radiological, histopathological, molecular characteristics, and treatment outcomes to further promote awareness of such a rare occurrence. Calvarial lymphoma is radiologically similar in many respects to meningioma, solid fibrous tumors, osteomyelitis, and metastatic carcinomas. Earlier in the course of the disease, the patient noticed a gradually enlarging scalp mass, which is the most common symptom. We believe that calvarial lymphoma tends to initially grow extracranially. It involves the bone and subcutaneous space before it can breach the dura [3-7]. This growth pattern differentiates calvarial lymphoma from lesions that initially involve the dura, such as meningioma. Additionally, due to the infiltrative growth of calvarial lymphoma through the diploic spaces and emissary veins, sclerotic bone changes can be seen on CT images at an early stage of the disease. Later on, bone assumes an osteolytic appearance, albeit mild, compared to the size of the lesion [8-12]. At the time of presentation, calvarial lymphoma would mostly have involved the extracranial as well as the intracranial spaces, which many authors consider to be more predictive of calvarial lymphoma [5]. Calvarial lymphoma on MRI shows variable signal intensity changes; however, upon gadolinium administration, it frequently exhibits classical enhancement on T1 images, as in our case [4]. This enhancement, although suggestive of calvarial lymphoma, is still nonspecific, as other dural-based tumors may still show the same pattern of enhancement. When calvarial lymphoma is suspected, it is important to look for systemic involvement and to supplement the examination with a CT scan of the chest and abdomen. Furthermore, a positron emission tomography scan [PET], when available, is also an indispensable tool in the evaluation and prognostication of systemic involvement. Confirmation of systemic involvement may require a bone marrow biopsy as well.

A meta-analysis conducted by Toyota and his colleagues showed that chemotherapy and radiation achieve high remission rates whether or not a prior surgery was undertaken [1]. The current consensus in the management of calvarial lymphoma is to obtain a biopsy followed by chemotherapy and/or radiotherapy [5].

Unfortunately, many of the reported cases in the literature have been treated with partial or complete resection. This practice has been mostly abandoned nowadays and is not backed by substantial evidence from the literature [2]. When a large surgical resection is planned pre-operatively, it’s important to have a frozen section available early in the surgery to avoid further exposing the patient to the inherent risks of surgery such as bleeding from the dural sinuses.

## Conclusions

When considering a patient with a progressively enlarging skull mass, it is important to include calvarial lymphoma in the differential diagnosis, despite its rarity. Awareness of this uncommon condition and maintaining a high level of suspicion help prevent the potential risks associated with open surgery and enable prompt evaluation of the patient for chemotherapy. This approach facilitates the timely initiation of chemotherapy and/or radiation therapy at an early stage.

## References

[1] Toyota E, Taslimi S, Alkins R (2021). Optimal management of calvarial lymphoma: a meta-analysis. World Neurosurg.

[2] El Asri AC, Akhaddar A, Baallal H (2012). Primary lymphoma of the cranial vault: case report and a systematic review of the literature. Acta Neurochir (Wien).

[3] Salvo V, Brogna B, Sampirisi L, Casinelli A, Emanuela R (2018). Diffuse-primary-B-cell lymphoma of the cranial vault presenting as stroke. Radiol Case Rep.

[4] Lee SH, Yun SJ (2018). Early stage primary cranial vault lymphoma in a 50-year-old man: presenting as only sclerosis and mimicking osteoma. Ann Hematol.

[5] da Rocha AJ, da Rocha TM, da Silva CJ, Paes RP, Bruniera P, Chiattone CS (2010). Cranial vault lymphoma: a systematic review of five patients. J Neurooncol.

[6] Maruyama D, Watanabe T, Beppu Y (2007). Primary bone lymphoma: a new and detailed characterization of 28 patients in a single-institution study. Jpn J Clin Oncol.

[7] Kanaya M, Endo T, Hashimoto D (2017). Diffuse large B-cell lymphoma with a bulky mass in the cranial vault. Int J Hematol.

[8] Edward Graveson Uff C, Louis Shieff C (2009). Massive transcalvarial lymphoma. BMJ Case Rep.

[9] Evliyaoğlu C, Ilbay K, Ercin C, Ceylan S (2006). Primary cranial vault lymphoma presenting as a traumatic subdural hematoma. Zentralbl Neurochir.

[10] Pardhanani G, Ashkan K, Mendoza N (2000). Primary non-Hodgkin's lymphoma of the cranial vault presenting with unilateral proptosis. Acta Neurochir (Wien).

[11] Tashiro R, Kanamori M, Suzuki H, Utsunomiya A, Meguro K, Uenohara H, Tominaga T (2015). Diffuse large B cell lymphoma of the cranial vault: two case reports. Brain Tumor Pathol.

[12] Fukushima Y, Oka H, Utsuki S, Nakahara K, Fujii K (2007). Primary malignant lymphoma of the cranial vault. Acta Neurochir (Wien).

